# Movement and Other Neurodegenerative Syndromes in Patients with Systemic Rheumatic Diseases: A Case Series of 8 Patients and Review of the Literature

**DOI:** 10.1097/MD.0000000000000971

**Published:** 2015-08-07

**Authors:** Rikitha Menezes, Alexander Pantelyat, Izlem Izbudak, Julius Birnbaum

**Affiliations:** From the Division of Rheumatology, The Johns Hopkins University School of Medicine, Baltimore, Maryland (RM); Department of Neurology, The Johns Hopkins University School of Medicine, Baltimore, Maryland (AP); Division of Neuroradiology, The Johns Hopkins University School of Medicine, Baltimore, Maryland (II); and Division of Rheumatology and Department of Neurology, The Johns Hopkins University School of Medicine, Baltimore, Maryland (JB).

## Abstract

Patients with rheumatic diseases can present with movement and other neurodegenerative disorders. It may be underappreciated that movement and other neurodegenerative disorders can encompass a wide variety of disease entities. Such disorders are strikingly heterogeneous and lead to a wider spectrum of clinical injury than seen in Parkinson's disease. Therefore, we sought to stringently phenotype movement and other neurodegenerative disorders presenting in a case series of rheumatic disease patients. We integrated our findings with a review of the literature to understand mechanisms which may account for such a ubiquitous pattern of clinical injury.

Seven rheumatic disease patients (5 Sjögren's syndrome patients, 2 undifferentiated connective tissue disease patients) were referred and could be misdiagnosed as having Parkinson's disease. However, all of these patients were ultimately diagnosed as having other movement or neurodegenerative disorders. Findings inconsistent with and more expansive than Parkinson's disease included cerebellar degeneration, dystonia with an alien-limb phenomenon, and nonfluent aphasias.

A notable finding was that individual patients could be affected by cooccurring movement and other neurodegenerative disorders, each of which could be exceptionally rare (ie, prevalence of ∼1:1000), and therefore with the collective probability that such disorders were merely coincidental and causally unrelated being as low as ∼1-per-billion. Whereas our review of the literature revealed that ubiquitous patterns of clinical injury were frequently associated with magnetic resonance imaging (MRI) findings suggestive of a widespread vasculopathy, our patients did not have such neuroimaging findings. Instead, our patients could have syndromes which phenotypically resembled paraneoplastic and other inflammatory disorders which are known to be associated with antineuronal antibodies. We similarly identified immune-mediated and inflammatory markers of injury in a psoriatic arthritis patient who developed an amyotrophic lateral sclerosis (ALS)-plus syndrome after tumor necrosis factor (TNF)-inhibitor therapy.

We have described a diverse spectrum of movement and other neurodegenerative disorders in our rheumatic disease patients. The widespread pattern of clinical injury, the propensity of our patients to present with co-occurring movement disorders, and the lack of MRI neuroimaging findings suggestive of a vasculopathy collectively suggest unique patterns of immune-mediated injury.

## INTRODUCTION

Movement and other neurodegenerative syndromes (ie, amyotrophic lateral sclerosis [ALS]) are associated with early mortality, a high rate of psychosocial morbidities (ie, risk of depression), osteoporotic fractures, wheelchair-dependence, and bulbar dysfunction (requiring dependence on percutaneous endoscopic gastrostomy (PEG) tubes and aspiration pneumonia).^1–3^ Aside from the association of antiphospholipid antibodies with chorea,^4^ the relationship of movement and other neurodegenerative disorders with different rheumatic diseases remains uncertain.

There are 2 challenges in the clinical approach to movement and other neurodegenerative disorders in rheumatic disease patients. First, just as inflammatory arthropathies encompass more than 20 different diseases with distinct clinical patterns and etiopathogenic mechanisms, there is similar although underappreciated heterogeneity which is associated with movement disorders. For example, Parkinson's disease is well recognized when presenting with tremor, rigidity, bradykinesia, and postural instability/gait difficulty.^5^ In contrast, there is a wider spectrum of Parkinsonian syndromes (ie, to be distinguished from Parkinson's disease), which may present with bradykinesia and rigidity without tremor, demonstrate early and severe postural instability, exhibit lack of response to dopaminergic therapy, have more rapid deterioration, and culminate with a wider clinical profile of findings not seen in Parkinson's disease.^6,7^ Such Parkinsonian syndromes may be associated with dementia, visual hallucinations, aphasia, cerebellar ataxia, dysautonomia, dystonia, and an alien-limb phenomenon.^8,9^ Therefore, a primary clinical challenge is to have an intimate familiarity with such movement disorders which may be misdiagnosed as Parkinson's disease.

A second challenge in the evaluation of movement and other neurodegenerative disorders is to ascertain whether such disorders are merely coincidental, noninflammatory, and not causally related to the background rheumatic disease—or whether such movement or other neurodegenerative disorders may be driven by immune-mediated mechanisms. Therefore, a scrupulous neurological examination, knowledge of disease prevalence, elucidation of atypical features, familiarity with disease heterogeneity, identification of immune-mediated correlates, and understanding of disease cadence may help in this challenging task of ascribing whether movement and other neurodegenerative disorders are idiopathic or due to rheumatic diseases.

In this manuscript, we therefore present a case series of rheumatic disease patients with movement and other neurodegenerative disorders. There were several interesting findings. First, we describe a wider spectrum of movement and other neurodegenerative disorders than previously reported in rheumatic disease patients. Second, a striking finding was that individual patients could present with co-occurring movement and other neurodegenerative disorders, each of which were individually rare, and having collective probabilities of ∼1-per-billion if merely coincidental, unrelated, and not unified by immune-mediated mechanisms. Finally, we provide the first example suggesting that an ALS-plus syndrome occurring in the context of tumor necrosis factor (TNF)-inhibitor therapy was immune-mediated and likely iatrogenically induced.

In addition to neurologists and rheumatologists, patients with these movement and other neurodegenerative disorders are cared for by physicians of different backgrounds—including family medicine practitioners and internists (caring for systemic manifestations of rheumatic diseases), gastroenterologists (deciding on the threshold for alimentary supplementation), psychologists and psychiatrists (due to high rates of depression), pulmonologists (caring for dyspnea due to neuromuscular weakness), and physiatrists (due to requirement for intensive physical therapy). Therefore, the integration of our case presentations with a systematic review of the literature suggests important mechanisms and therapeutic strategies which will be of interest to this multidisciplinary team of physicians.

## PREVIEW

### Introduction

For purposes of clarifying the terminology used in the case presentations, and for a full description of the symptoms and examination findings seen in the respective cases, we have provided a brief *preview* section (see below). Information reported in the case presentations was deidentified, and patients provided informed consent. Before reading the case presentations, the reader is encouraged to first read parts (A) and (B) of this preview section. Section (A) of this preview section defines important differences between the terms “Parkinson's disease,” “Parkinsonism,” and “atypical Parkinsonian syndromes.” Section (B) defines the term “neurodegenerative” disorders as used in this manuscript, and emphasizes that this is a clinical/radiographic designation and does not causally imply underlying mechanisms. Section (C) provides a full description of the symptoms and clinical findings associated with the various movement and other neurodegenerative disorders described in the case vignettes. Therefore, we recommend that Section (C) should optimally be read only in conjunction with the case presentations, and does not need to be read before the case vignettes.

### (A) Definitions and Distinction Between Parkinson's Disease, Parkinsonism, and Atypical Parkinsonian Syndromes

#### (a.1) Parkinson's Disease

This is a clinical diagnosis associated with bradykinesia (ie, slowed movements, and interchangeably referred to as “akinesis”), rigidity (which can be of the “cogwheeling” type), tremor, and postural instability.^10,11^

#### (a.2) Parkinsonism

This is a clinical designation of examination findings. The findings include bradykinesia, rigidity, tremor, and postural instability with a tendency to fall.

#### (a.3) Atypical Parkinsonian Syndromes

These refer to a wide variety of syndromes, which are differentiated from Parkinson's disease based on distinguishing symptoms, examination findings, and neuropathological features.^12^ Although frequently presenting with bradykinesia and rigidity (ie, termed as an “akinetic-rigid” syndrome), these atypical Parkinsonian syndromes characteristically lead to an earlier and more expansive pattern of injury compared to Parkinson's disease. Such syndromes may cause ataxia, dysautonomia, alien-limb phenomenon, and visual hallucinations.^8^ These atypical Parkinsonian syndromes may be misdiagnosed and misclassified as Parkinson's disease, and may respond poorly and/or in an unsustained manner to levodopa (l-dopa) therapy.^13^

### (B) Neurodegenerative Disorders

For purposes of this manuscript, the term “neurodegenerative” disorder refers to any syndrome in which there is objective magnetic resonance imaging (MRI) evidence showing degeneration of central nervous system (CNS) anatomic regions (ie, cerebellar degeneration), or in which there is objective electrodiagnostic or skin-biopsy evidence showing degeneration of peripheral nervous system (PNS) anatomic structures. In this manuscript, this designation of a neurodegenerative disorder is therefore a purely clinical term, and does not causally imply whether neuronal loss of PNS and/or CNS structures is due to a primary neurodegenerative process or due to immune-mediated mechanisms.

### (C) Symptoms and Clinical Findings Associated With Atypical Parkinsonian Syndromes and Neurodegenerative Disorders: CNS Syndromes

#### (c.1) Multiple System Atrophy

The syndrome of multiple system atrophy (MSA) refers to patients who can have prominent dysautonomia, Parkinsonism, and often early postural instability in the absence of a robust, sustained response to dopaminergic therapy.^14^ MSA leads to a more expansive pattern of CNS injury than seen in Parkinson's disease, may feature akinesis and rigidity in the absence of tremor (ie, an akinetic-rigid syndrome), and encompasses 2 entities: *MSA-Cerebellar type (MSA-C)*,^14–17^ which features Parkinsonism associated with prominent cerebellar findings; *MSA-Parkinsonian type (MSA-P)*,^17,18^ which features rapidly progressive Parkinsonism and can be associated with marked postural instability, axial dystonia with anterocollis, characteristic stridor, pyramidal/upper-motor-neuron findings (ie, hyperreflexia and Babinski responses), and with no/few cerebellar features.^14,16,18^

MSA may be stratified into tiers of diagnostic certainty (definite/probable/possible), with the gold standard of “definite” MSA based on neuropathological findings on postmortem examination.^17^ The graduation from possible to probable MSA is in part based on clinical severity and progression of dysautonomia.^17^ In routine clinical practice, a substantial proportion of patients initially encountered may have possible MSA, but need aggressive multidisciplinary care instituted even at this tier of diagnostic certainty.

#### (c.2) Corticobasal Syndrome (CBS)

This syndrome may include akinesis and rigidity with no sustained response to l-dopa therapy, and is associated with other asymmetric findings.^19^ Such prominent findings include asymmetric and unilateral limb “apraxia.” The term apraxia refers to the clinical phenomenon whereby patients may have normal limb strength, but because of cortical dysfunction lose the ability to use the limb to perform sequential motor tasks. CBS can be associated with an “alien-limb” phenomenon, in which patients articulate a sensation that the limb is functionally deafferented, does not feel like a part of the patient's body, and may be associated with involuntary movements.^20^ There can also be unilateral dystonia or other posturing of the affected limb(s), as well as myoclonus and cortical sensory loss.

#### (c.3) Progressive Supranuclear Palsy (PSP)

This disorder may present as an akinetic-rigid syndrome, and is appropriately named given that patients develop prominent, supranuclear gaze palsy.^21,22^ Downgaze is usually affected before impaired upgaze. In addition, patients may have prominent retropulsion (ie, falling backwards), postural instability, neck dystonia, and classically suffer from falls within 12 months of symptoms onset—which is significantly earlier compared to Parkinson's disease.^23^ Other supportive evidence includes onset at age 40 or later, and no evidence for other atypical Parkinsonian syndromes.^21^

#### (c.4) Cerebellar Degeneration

This refers to a disorder in which patients may experience a variety of symptoms and findings related to cerebellar dysfunction.^24^ This may include “midline” findings (ie, truncal instability), “appendicular” findings (ie, limb dysmetria), as well as abnormal, characteristic eye findings elicited on cranial-nerve examination. In this manuscript, the term cerebellar degeneration refers to such abnormal examination findings associated with neuroimaging findings of cerebellar degeneration, and does not imply or discriminate whether these features are due to a primary neurodegenerative process versus immune-mediated injury.

#### (c.5) Frontotemporal Dementia (FTD)

This refers to a “cortical” dementia which may affect the frontal and/or temporal lobes, and may present with behavioral changes and/or with language deficits.^25^ Impairment in language may cause predominantly effortful and nonfluent speech, and is referred to as progressive, nonfluent aphasia.^26^ In contrast, language comprehension may be affected, leading to a “fluent” aphasia termed as a semantic dementia.^27^ The behavioral variant of FTD may be associated with frontal lobe disinhibition. In the earliest stages of aphasia, more global impairment in other cognitive deficits may not be evident, and patients can initially have a normal mini-mental status examination (MMSE).^26,28^

### (D) Peripheral Nervous System (PNS) Syndromes Associated With Dorsal Root Ganglia Degeneration

#### (d.1) Sensory Neuronopathy

This is a PNS disorder associated with prominent loss of position sense and a sensory ataxia.^29,30^ The severity of proprioceptive impairment may be sufficient to relegate patients to wheelchairs, even when there is normal strength. Sensory neuronopathies are associated with neurodegeneration of large-sized dorsal root ganglia (DRG).^31,32^ Electrodiagnostic studies aid in the diagnosis of sensory neuronopathies and can also serve as mechanistic surrogates of DRG neurodegeneration.^29,30,32^

#### (d.2) Non-length-Dependent, Small-Fiber Neuropathies

This is a painful neuropathy which can affect smaller-sized, nociceptive, thinly myelinated A-delta fibers, and unmyelinated C-fibers.^33,34^ The pattern of neuropathic pain may be diffuse, and does not necessarily conform to a traditional, “stocking-and-glove” distribution. Skin-biopsy studies are diagnostic and also serve as mechanistic surrogates of small-sized DRG neurodegeneration.^33,34^

## CASE PRESENTATIONS

### Sensory Neuronopathies Associated With Cerebellar Degeneration

#### Case 1: Sensory Neuronopathy, Non-length-Dependent, Small-Fiber Neuropathy, Cerebellar Degeneration, and Frontotemporal Dementia in a Sjögren's Syndrome Patient

A 65-year-old, right-handed, Caucasian female was evaluated at our center for progressive gait instability, abnormal eye movements, limb ataxia, sensory neuronopathy, and a nonfluent aphasia occurring in the context of seronegative Sjögren's syndrome (SS). Her SS was characterized by a 6-year history of sicca symptoms, abnormal Schirmer's test (ie, showing decreased tear production), and a diagnostic lip biopsy revealing focal lymphocytic sialadenitis with a focus score of 1.2.^35^

**FIGURE 1:**
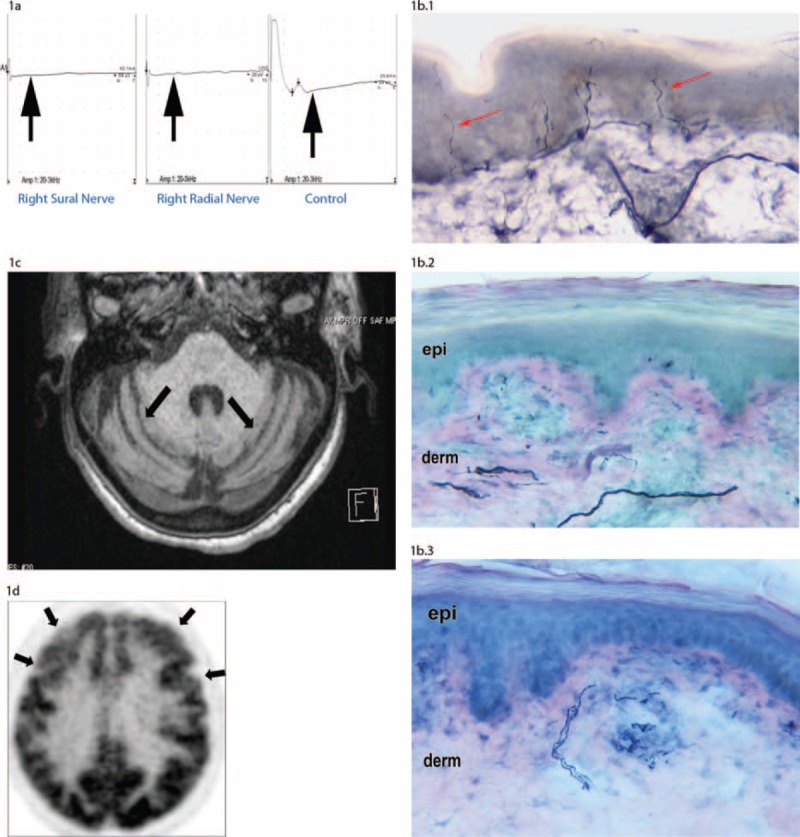
Nerve-conduction, skin-biopsy, and neuroimaging studies in a Sjögren's syndrome patient with co-occurring sensory neuronopathy, non-length-dependent, small-fiber neuropathy, cerebellar degeneration, and frontotemporal dementia. (A) Nerve-conduction studies: The sensory nerve action potentials (SNAPs) have a flat contour and are not elicited for the sural (left panel) or radial (middle panel) sensory nerves (arrows). Such diffuse loss of SNAPs is characteristic of a sensory neuronopathy, and is also an electrodiagnostic indicator of large-sized, DRG neurodegeneration.^31,32^ In contrast, the SNAPs from a control Sjögren's syndrome (SS) patient without a neuronopathy (right panel) are easily elicited. (B) Skin-biopsy studies: Skin-biopsy studies are diagnostic for a small-fiber neuropathy when there is decreased intraepidermal nerve-fiber density of unmyelinated nerves. Unmyelinated C-fiber nerves are immunostained against the panaxonal protein PGP 9.5. In (B.1) of an SS patient without a small-fiber neuropathy, there is normal, intraepidermal nerve-fiber density of unmyelinated nerves (arrow). In contrast, (B.2) and (B.3) are reflective of skin-biopsy specimens which are diagnostic of a non-length-dependent, small-fiber neuropathy. Compared to the normal SS control, there is markedly decreased intraepidermal nerve-fiber density in biopsies taken from the proximal thigh (B.2), as well as the distal leg (B.3). This pattern of decreased intraepidermal nerve-fiber density in both the proximal thigh as well as the distal leg is a surrogate marker of small-sized DRG neurodegeneration.^33,34^ (C) MRI studies: On a T1 axial image, enlargement of the cerebellar sulci is consistent with cerebellar degeneration.(D) PET studies of the brain: On an axial PET image, hypometabolism in bilateral frontoparietal cortical regions is consistent with frontotemporal dementia.

In the 5 years before evaluation at our center, the patient developed diffuse neuropathic pain, gait impairment, and frequent falls. Her workup revealed 2 subtypes of related SS PNS syndromes which are associated with DRG neurodegeneration (see Preview Sections d.1 and d.2). First, she had a PNS disorder referred to as a sensory “neuronopathy,” which is associated with large-sized DRG neurodegeneration^29–32^ (see Preview Section d.1). Examination findings included diffuse hyporeflexia, loss of position sense in the feet, and a sensory ataxia. Electrodiagnostic findings which were diagnostic of a neuronopathy and are also surrogate markers of large-sized DRG neurodegeneration included diffuse absence of all sensory nerve action potentials (SNAPs)^29–32^ (Figure 1A).

Second, she had a sensory neuropathy referred to as a “non-length-dependent,” small-fiber neuropathy^33,34^ (see Preview Section d.2), with widespread neuropathic pain experienced in the face, arms, and lower extremities. Her neurological examination revealed diffuse deficits to “small-fiber” modalities (ie, pinprick and temperature) in symptomatic regions. Skin-biopsy findings were both diagnostic of a small-fiber neuropathy, and occurring in a pattern consistent with small-sized DRG neurodegeneration^29,30^ (Figure 1B).

In the 18 months before evaluation at our center, she complained of increasing imbalance, suffered numerous falls despite using a walker, and was relegated to a wheelchair. On our examination, in addition to having the above-described PNS syndromes associated with DRG neurodegeneration, she also had superimposed clinical and imaging findings associated with cerebellar degeneration and which were contributing to her falls (see Preview Section c.4). Specifically, she had abnormal eye findings attributable to cerebellar disease (slowed downward saccades, choppy smooth pursuits, and an abnormal vestibulo-cochlear reflex with decreased gain upon head movement); dysmetria and ataxia in the upper limbs (due to cerebellar disease); and ataxia in the lower limbs (due to both cerebellar disease and sensory ataxia). Indeed, her MRI neuroimaging studies noted cerebellar atrophy (Figure 1C). There was no evidence of paraneoplastic-associated antibodies (ie, no anti-Hu antibodies),^36^ celiac disease^37,38^ (ie, negative antitransglutaminase IgG/IgA antibodies), and other syndromes which may jointly cause cerebellar degeneration and a sensory neuronopathy. Her workup also did not reveal other infectious (ie, HIV), metabolic (ie, copper and vitamin B12 deficiency), inflammatory (ie, demyelinating disease), or toxic exposures (ie, alcoholism) as causes of cerebellar degeneration.

Over the next year, she then further developed a progressive, nonfluent aphasia with normal comprehension. At this time, her MMSE was a 27/30. A positron emission tomography (PET) scan revealed bilateral hypometabolism in the frontal and temporal cortices, which was consistent with frontotemporal dementia (FTD) (Figure 1D). Such PET findings can identify FTD in patients presenting with this subtype of a progressive nonfluent aphasia (ie, as seen in Patient 1), even when the MMSE is initially not indicative of more diffuse cognitive deficits.^26,28^

Although sensory neuronopathies and small-fiber neuropathies in SS have been reported to selectively respond to intravenous immunoglobulin (IVIg),^39–42^ such IVIg therapy was contraindicated given a prior history of transient ischemic attacks. Immunosuppressive therapy was considered to be contraindicated given that she had experienced repeated episodes of life-threatening pneumonia and urosepsis requiring multiple admissions to the Intensive Care Unit.

The respective frequencies of these individual syndromes are rare: including the sensory neuronopathies (prevalence of ∼1:1000 in SS patients),^29,42,43^ cerebellar degeneration (prevalence of <1:1000 in SS patients),^43^ and FTD (prevalence of ∼8:10,000).^44^ Therefore, the collective likelihood that these disorders jointly occurred by random chance, and were not mechanistically interrelated, has a probability of ∼1-per-billion. Therefore, shared immune-mediated mechanisms which may account for the clustering of these disorders in a single patient are further considered in the Discussion Section.

#### Case 2: Sensory Neuronopathy, Non-length-Dependent, Small-Fiber Neuropathy, and Cerebellar Degeneration in an SS Patient

A 65-year-old, right-handed, Caucasian gentleman was referred for evaluation of gait instability occurring in the context of SS. The patient's SS was characterized by a 3-year history of sicca symptoms, abnormal parotid scintigraphy, and a lip biopsy revealing focal lymphocytic sialadenitis with a focus score of 2.3. In the 5 years before evaluation at our center, the patient experienced diffuse neuropathic pain associated with gait imbalance and falls. Two years before evaluation, such progressive gait deterioration led to dependence on a walker.

Prior neuroimaging studies of the brain revealed nonspecific white matter disease. Initial electrodiagnostic studies did not reveal any denervating changes and were thought to be consistent with a sensory neuropathy.

**FIGURE 2:**
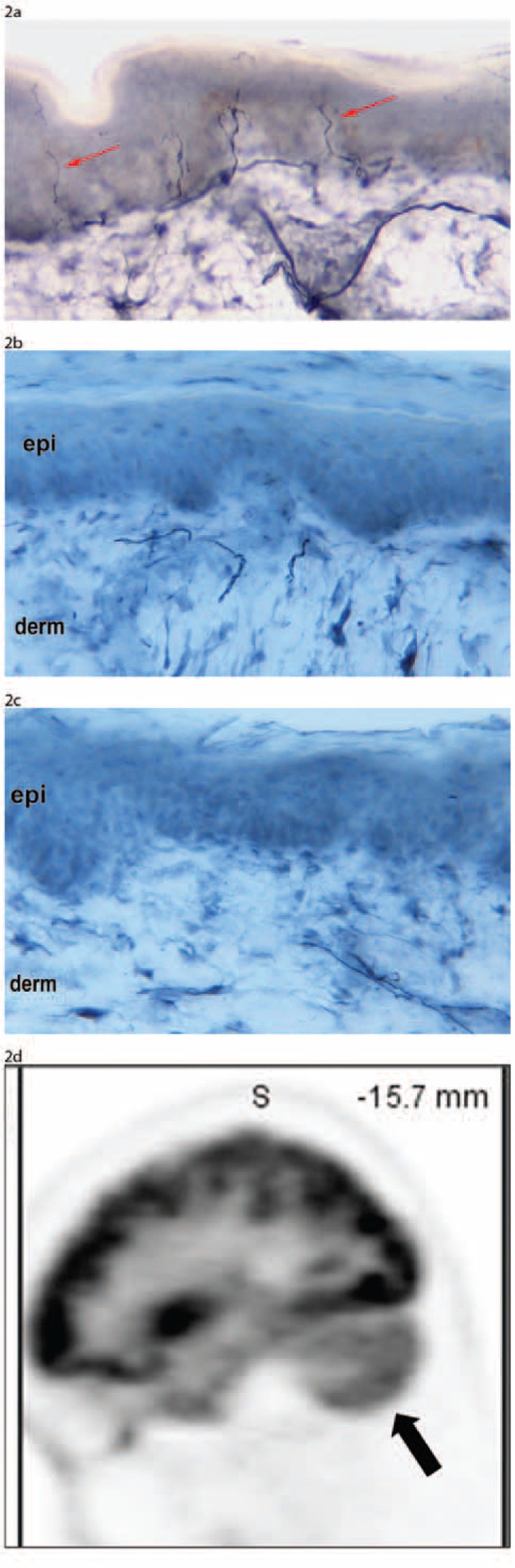
Skin-biopsy and PET imaging in a Sjögren's syndrome (SS) patient with co-occurring sensory neuronopathy, non-length-dependent, small-fiber neuropathy, and cerebellar degeneration. (A–C) Skin-biopsy studies: Similar to Figure 1, this SS patient had skin-biopsy studies diagnostic of a non-length-dependent, small-fiber neuropathy. Compared to the normal SS control without a small-fiber neuropathy (A), there is decreased intraepidermal nerve-fiber density of unmyelinated nerves in both the proximal thigh (B) and the distal leg (C). This pattern is also a surrogate marker of small-sized DRG neurodegeneration.(D) PET studies: On sagittal PET image, hypometabolism in the cerebellum is noted.

Neurological examination at our center revealed abnormal cerebellar findings, including evidence of “cerebellar” eye disease (ie, abnormal, direction-changing nystagmus, abnormal saccades, and abnormal smooth muscle pursuits), associated with truncal and limb ataxia. Similar to the above-described Patient 1, he also had evidence of a sensory neuronopathy as well as a non-length-dependent, small-fiber neuropathy. His electrodiagnostic and skin-biopsy studies were diagnostic of these respective PNS syndromes, and were also surrogate indicators of DRG neurodegeneration (Figure 2A–C).^29–34^ In association with cerebellar examination findings, MRI neuroimaging studies now showed cerebellar atrophy. Due to symptoms of dry cough and a 20-year history of tobacco use, there was concern about an occult malignancy. The patient therefore underwent computed tomography (CT) and subsequent PET scans which did not demonstrate any hypermetabolic foci, but interestingly did demonstrate cerebellar hypometabolism (Figure 2D). Given that both sensory neuronopathies and nonlength-dependent, small-fiber neuropathies in SS may respond to IVIg, we are trying to obtain insurance approval for IVIg therapy.

### Progressive Supranuclear Palsy

#### Case 3: Progressive Supranuclear Palsy (PSP) Associated With Corticobasal Syndrome (CBS) in an SS Patient

An 80-year-old, right-handed, Caucasian female was referred for evaluation of a presumed diagnosis of Parkinson's disease occurring in the context of SS. Her SS was characterized by a 7-year history of sicca symptoms, decreased tear production on Schirmer's test, and anti-Ro/SS-A antibodies. In the 2 years before evaluation at our center, she developed rapid onset of gait deterioration, experienced multiple falls within only 3 months of onset of these gait symptoms, and was predominantly relegated to a wheelchair within 1 year. In addition, she verbalized a sensation that “my right-hand does not belong to me.” She developed impaired fine-motor dexterity, and ultimately lost the ability to use her right hand for routine functional tasks. She was given the diagnosis of Parkinson's disease, but had no improvement on l-dopa therapy (100 mg, 3 times daily). MRI neuroimaging of the brain was unremarkable.

She presented to our clinic in a wheelchair. Her examination was notable for bradykinesia and cogwheel rigidity in the absence of tremor, and she intriguingly had additional findings which were consistent with both CBS and PSP (see Preview Sections c.2 and c.3).^22,45^ With regard to CBS, she had a right “alien-limb” syndrome, with unilateral, limb apraxia, and dystonia. For example, despite normal strength in her right upper-extremity, she was unable to use her right hand for any tasks and there was abnormal limb posturing.

With regard to PSP, she had impaired upgaze and downgaze, postural instability, was only able to walk 5 yards with 1-person assistance, had an unsteady festinating gait with decreased arm-swing and required 6 steps to turn.

Given that the patient had already been affected by severe and cumulative disability (early falls and wheelchair-dependence consistent with PSP, severity of limb apraxia and alien-limb syndrome consistent with CBS), she did not wish to be empirically treated with immunosuppressive therapy.

These individual syndromes are rare, with PSP having a prevalence of only ∼6/100,000^21,46^ and CBS having a prevalence of ∼7/100,000.^47^ Both syndromes may reflect a similar spectrum of neuropathological changes,^20,48^ and the propensity of both syndromes to co-occur in a single patient is further considered in the Discussion Section.

#### Case 4: PSP in an SS Patient

An 82-year-old, right-handed, Caucasian gentleman was referred for a presumed diagnosis of Parkinson's disease occurring in the context of SS. His SS was characterized by a 2-year history of sicca symptoms, decreased tear production on Schirmer's test, and anti-Ro/SS-A antibodies. In the 2 years before evaluation at our center, he developed rapid onset of gait deterioration, experienced multiple falls within 3 months after onset of symptoms, suffered a hip fracture, developed difficulty with transfers, had complications from sacral decubitus ulcers, was relegated to a wheelchair after 18 months, and ultimately needed to be placed in an assisted-living facility. The patient had received a prior diagnosis of Parkinson's disease but had no improvement on l-dopa therapy.

His examination was notable for bradykinesia and cogwheel rigidity in the absence of tremor, and had additional findings which were consistent with PSP^21,22^ (Preview Section c.3). He had impaired upgaze and downgaze, postural instability, and was wheelchair-bound and unable to walk even with assistance. Neuroimaging of the brain revealed only nonspecific white-matter disease.

Given his extensive functional debility and recurrent sacral decubitus ulcers, treatment with immunomodulatory therapy was deferred.

### Multiple System Atrophy

#### Case 5: Multiple System Atrophy of the Parkinsonian Type in a Patient With Undifferentiated Connective Tissue Disease

A 63-year-old, right-handed, Caucasian gentleman was referred for further evaluation of a Parkinsonian syndrome occurring in the context of a rheumatic disease. Three years before evaluation at our center, he developed an unsteady gait, MRI of the cervical spine showed mild compression at C3 to C4, and he underwent anterior fusion of C3 to C4. However, he continued to have further gait deterioration.

**FIGURE 3:**
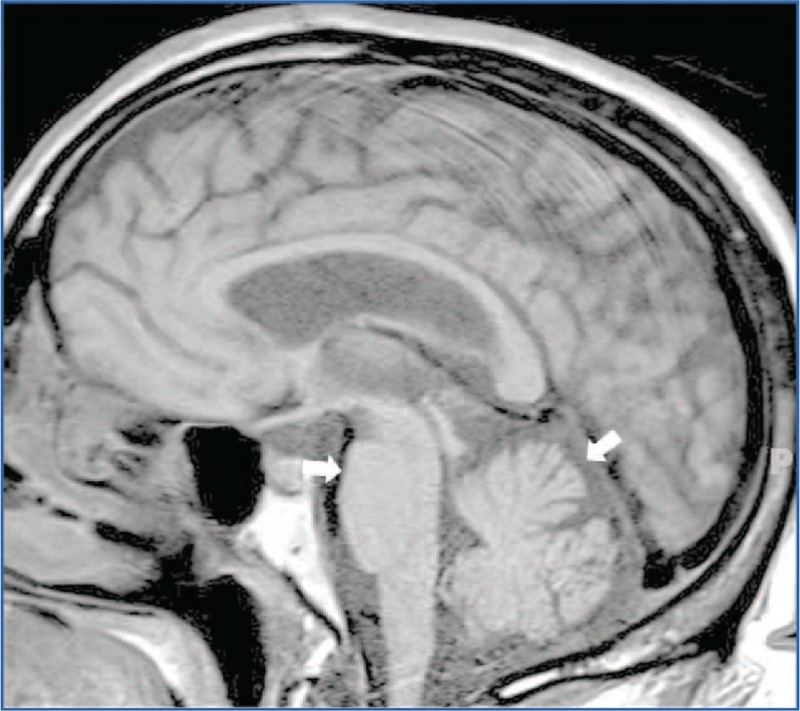
MRI findings in a patient with Multiple System Atrophy—Parkinsonian type. Sagittal view of the brain on MRI FLAIR sequence demonstrates that there is diffuse atrophy of the pons, cerebellum, and midbrain.

At this time, neurological evaluation noted bradykinesia and cogwheel rigidity in the absence of resting tremor, a presumptive diagnosis of Parkinson's disease was entertained, but there was no improvement on l-dopa therapy (600 mg, 3 times daily). MRI revealed prominent medullary, pontine, and cerebellar atrophy (Figure 3), and the diagnosis of MSA was considered (Preview Section c.1). Over the 18 months before evaluation at our center, he developed urogenital dysfunction ultimately requiring placement of an indwelling Foley catheter, suffered increased falls, and was relegated to a wheelchair.

In the year before evaluation at our center, he was diagnosed with undifferentiated connective tissue disease (UCTD), characterized by new-onset of Raynaud's, polyarthalgias, and with serologies notable for anti-CCP antibodies (low-titer) without synovitis and anti-Pm1-Scl antibodies. On evaluation at our center, he presented with findings consistent with UCTD, including puffy hands without synovitis, no sclerodactyly, no digital pit ulcers, mildly dilated nailfold capillaries, and no proximal weakness. On neurological assessment, he presented in a wheelchair and with an indwelling Foley catheter. He was noted to have an akinetic-rigid syndrome without tremor, torticollis and anterocollis of his neck, movements causing flexion of his trunk, and was unable to walk independently. His rapid progression of Parkinsonism without resting tremor, falls, refractoriness to both surgical intervention and l-dopa therapy, urogenital dysfunction, and absence of PSP findings (ie, supranuclear gaze palsy), were most consistent with MSA of the Parkinsonian type; ie, MSA-P (Preview Section c.1).^18^ Given his severe functional debility, the patient and his family wished to defer immunomodulatory therapy.

#### Case 6: MSA-P in an SS Patient

A 76-year-old, right-handed, Caucasian female was referred for evaluation of gait impairment occurring in the context of SS. Her SS was characterized by a decade of sicca symptoms, decreased tear production on Schirmer's test, and anti-Ro/SS-A antibodies. In the 6 months before evaluation at our center, she developed rapid gait deterioration, experienced multiple falls, and required a wheelchair when exiting her home. Her neurological examination at our center was consistent with MSA-P (Preview Section c.1).^18^ There was urogenital dysfunction leading to multiple urinary tract infections, absence of tremor with bradykinesia and cogwheel rigidity, a festinating gait with inability to take more than 10 steps without support, and upper-motor findings (ie, hyperreflexia and Babinski findings) without structural disease of the brain or spinal cord on MRI neuroimaging studies. Although the rapidity of her gait deterioration was somewhat atypical of “idiopathic” MSA-P (in which the time between disease onset and wheelchair dependency is usually ∼ 3–7 years),^18,49^ there was no evidence of supranuclear gaze palsy, increased axial tone, or other findings which were more suggestive of PSP. She did not wish to try immunomodulatory therapy.

### Hemi-Parkinsonism

#### Case 7: Hemi-Parkinsonism in a Patient With Undifferentiated Connective Tissue Disease

A 46-year-old, left-handed, Caucasian gentleman was referred for evaluation of presumptive Parkinson's disease occurring in the context of UCTD. The patient initially developed “stiffness” of the left shoulder, along with development of a resting tremor of the left hand. Subsequently, over a 1-year period, he developed a synchronous resting unilateral tremor, which completely spared the right limbs, affected the hands and larger joints in the left upper-extremity, and synchronously occurred as a large-amplitude tremor across the left hip and knee joints. There was also fine-motor impairment on the left-side. There was no improvement with l-dopa therapy (250 mg, 3 times daily).

The patient had a known prior diagnosis of UCTD which was characterized by recurrent aphthous ulcerations, polyarthralgias (without synovitis), 5 years of sicca symptoms (but without anti-Ro/SS-A and anti-La/B antibodies, and with a lip biopsy not supportive of SS), and labs showing low-titer anti-CCP antibodies. Further evaluation revealed that the patient had medium-titer beta-2-glycoprotein IgM and IgG antibodies on serial occasions, with no anticardiolipin antibodies or lupus anticoagulant, no abnormal skin findings (ie, livedo-reticularis) or history of thrombotic disease, and normal neuroimaging studies of the brain.

The patient was referred to our center 2 years after onset of neurological symptoms. Neurological evaluation revealed bradykinesia, a resting, 3-Hz tremor which was strikingly synchronous and concomitantly affected the left arm and left leg, and occasional left-sided lower-extremity dystonia. Given atypical features of his movement disorder, the patient was diagnosed as having a hemi-Parkinsonian syndrome occurring in the context of UCTD.

Given recent studies suggesting that both thrombotic and nonthrombotic manifestations of antiphospholipid syndrome may respond to rituximab,^50–52^ our initial preference was to initiate rituximab therapy. However, his insurance company denied coverage for rituximab. The patient was treated with 6 months of intravenous cyclophosphamide at 750 mg/m^2^, followed by mycophenolate mofetil at 2000 mg per day for 6 months. There has been no improvement in his hemi-Parkinsonian symptoms, and attempts to obtain insurance approval for IVIg or rituximab are ongoing.

### Amyotrophic Lateral Sclerosis

#### Case 8: ALS in a Patient With Psoriatic Arthritis Treated With Tumor Necrosis Factor (TNF)-Inhibitor Therapy

A 56-year-old, right-handed, Caucasian female with a history of psoriatic arthritis was referred for evaluation of ALS. The patient was diagnosed with psoriatic arthritis at the age of 47, when she presented with psoriasis and a symmetric polyarthritis affecting the hands and feet. Her polyarthritis was refractory to nonbiological, Disease-Modifying Antirheumatic drugs (DMARDs), including methotrexate and leflunomide. Two years before evaluation, TNF-inhibitor therapy was initiated with infliximab at 5 mg/kg, which led to resolution of her polyarthritis. However, 1 year after infliximab therapy, over the span of only 4–6 weeks, she developed acute onset of tetraparesis, resulting in more than 20 falls. Neuroimaging studies of the brain and entire spine were unremarkable.

Initial clinical evaluation revealed mixed lower and upper-neuron findings suggestive of ALS. Electrodiagnostic studies were interpreted as confirming the diagnosis of ALS,^53^ which included diffuse acute and chronic 3+ to 4+ denervating changes in the trunk, upper extremities, and lower extremities. Infliximab was discontinued, but the patient had worsening weakness. Her gait deteriorated, to the extent that she constantly required a walker.

The patient was referred to our center 1 year after onset of neurological symptoms. Upon evaluation, she was noted to have diffuse muscle atrophy, dysarthria with complaints of dysphagia, barely preserved antigravity strength in her limbs (Medical Research Council 3/5), 3+ to 4+ patellar and upper-extremity reflexes but only 1+ Achilles reflexes, bilateral Babinski findings, and was wheelchair-bound.

Repeat electrodiagnostic studies revealed an unchanged pattern of acute denervating changes in the upper extremities, the trunk, and the lower extremities, and electromyography (EMG) of the bulbar musculature was not performed. However, nerve conduction studies revealed atypical findings for motor neuron disease, with markedly decreased peroneal compound motor action potential (CMAP) but with normal tibial CMAP, and with an axonal sensory polyneuropathy characterized by mildly reduced bilateral sural, median, and ulnar SNAPs. There were other atypical findings for ALS which were subsequently elicited on further serological and immunological studies. Specifically, she was noted to have antineuronal antibodies against the P/Q-type calcium channel. Given that this antibody can be associated with paraneoplasia,^54,55^ a CT scan of the chest/abdomen/pelvis was performed but did not reveal evidence of an occult malignancy. The patient also did not have clinical syndromes which have been otherwise reported in association with this antibody, such as Lambert–Eaton syndrome.^56^

In addition, lumbar puncture studies showed strikingly elevated total protein of 117 mg/dl in the absence of pleocytosis. Therefore, given the cumulative manifestations which were atypical for ALS: including acute onset of weakness, clinical and electrodiagnostic findings of a sensory polyneuropathy, the presence of antineuronal antibodies, and markedly elevated cerebrospinal fluid (CSF) total protein level, we recommended that she be admitted for in-patient plasmapheresis or IVIg. Due to overall functional debility, worsening dysarthria with complaints of dysphagia, clinicians and the patient considered the risks of immunosuppressive therapy to be prohibitive. Over the ensuing months, attempts to obtain approval for IVIG or plasmapheresis were unsuccessful. At time of last evaluation 4 months later, she was in a nursing home, had worsening quadraparesis with loss of antigravity strength, was unable to independently transfer, and required constant care for all activities of daily living.

Given these atypical findings for ALS, this patient is subsequently referred to as having an “ALS-plus” syndrome throughout the remainder of this manuscript.

## RESULTS

### Clinical Findings, Diagnostic Assessment, Therapeutic Interventions, and Outcomes

Table 1 summarizes our patients’ presentations, including the underlying rheumatic diseases, the clinical presentations of movement and other neurodegenerative disorders, as well as atypical features not usually encountered in the context of idiopathic and noninflammatory disorders. Altogether, our patients presented with a wide spectrum of Parkinsonian syndromes, which could be mistaken for Parkinson's disease. These included patients with PSP (Patients 3–4), CBS (Patient 3), and MSA-P (Patients 5–6). Other clinical syndromes included cerebellar degeneration (Patients 1–2), FTD (Patient 1), hemi-Parkinsonism (Patient 7), and an ALS-plus syndrome (Patient 8).

**TABLE 1:**
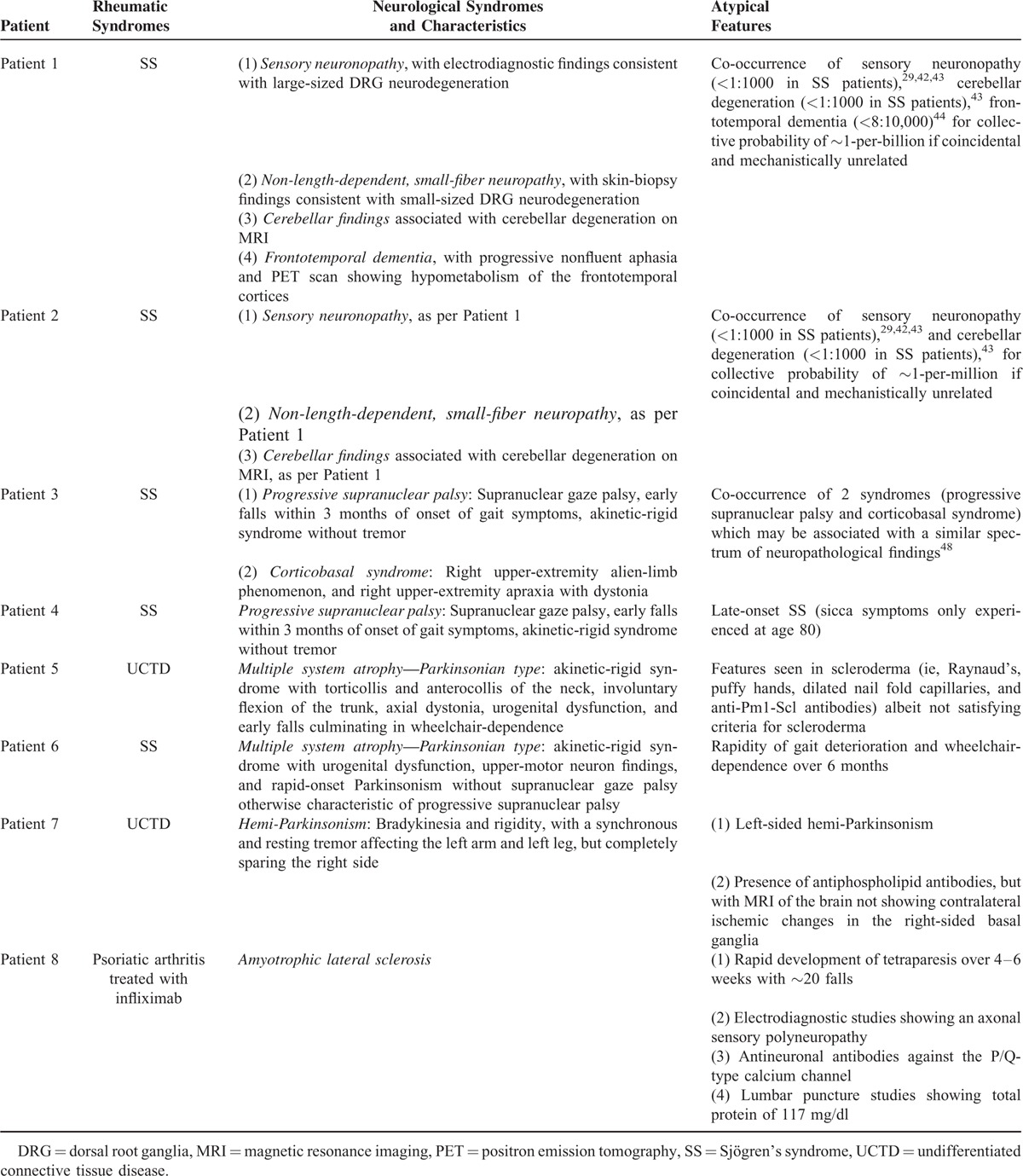
Summary of Clinical Characteristics, Ancillary Studies, and Atypical Features of Movement and Other Neurodegenerative Disorders Described in This Case Series

There were 3 patients with PNS disease. Two patients experienced sensory neuronopathies as well as non-length-dependent, small-fiber neuropathies (Patients 1–2), which are respectively associated with large-size and small-size DRG neurodegeneration. The patient with a TNF-inhibitor-associated ALS-plus syndrome also atypically had an axonal sensory polyneuropathy.

These collective syndromes caused a wide spectrum of clinical findings, including postural instability with early falls, alien-limb phenomenon with limb apraxia, unilateral Parkinsonism, truncal and neck dystonia, abnormal eye movements, and nonfluent aphasia. In the 2 patients respectively presenting with sensory neuronopathies and nonlength-dependent, small-fiber neuropathies (Patients 1–2), there was marked loss of joint position, sensory ataxia, and diffuse neuropathic pain not conforming to a “stocking-and-glove” distribution.

Of the underlying rheumatic diseases, the most common was SS, diagnosed in 5 patients. There were 2 patients with UCTD, including a single patient who interestingly presented with some features seen in scleroderma (ie, Raynaud's, puffy hands, dilated nailfold capillaries, and anti-Pm1-Scl antibodies) albeit not satisfying diagnostic criteria for scleroderma.^57^ There was a single patient with psoriatic arthritis treated with TNF-inhibitor therapy.

A particularly striking finding was that several patients presented with co-occurring clinical features of multiple neurodegenerative disorders. As illustrated in Table 1, the prevalence of these respective disorders was sufficiently rare such that the cooccurrence of these neurodegenerative syndromes presenting by random chance had probabilities ranging from ∼1-per-million to ∼1-per-billion. For example, Patient 1 presented with a non-length-dependent, small-fiber neuropathy, a sensory neuronopathy (prevalence of 1-per-1000 in SS),^29^ clinical and MRI evidence of cerebellar degeneration (prevalence of <1:1000 in SS), and FTD characterized by progressive nonfluent aphasia and with a PET scan showing hypometabolism of the frontal and temporal cortices (prevalence of 8 per 10,000).^44^ Similarly, the co-occurrence in SS Patient 2 of a non-length-dependent, small-fiber neuropathy, a sensory neuronopathy, and cerebellar degeneration had a collective likelihood of ∼1-per-million if occurring by chance.

Patient 3 was an SS patient who simultaneously presented with clinical features of both PSP and CBS. Interestingly, the clinical syndromes of PSP and CBS may reflect a continuum of neuropathological changes, and may account for why there may be overlapping clinical findings which are shared between these clinical syndromes.^20,48^ For example, our patient presented with unilateral limb apraxia and supranuclear gaze palsy, which is seen in both CBS and PSP.

In our patients with widespread clinical findings which ubiquitously suggested damage to both PNS (ie, DRG) and different CNS anatomic tracts (ie, cerebellum and frontotemporal cortices) one potential mechanism is a diffuse vasculopathy. However, none of our patients presented with MRI studies showing radiographic patterns of diffuse vascular disease. Patient 7 presented with a left-sided hemi-Parkinsonian syndrome, was the only patient who had antiphospholipid antibodies, and therefore was the only patient with clinical and immunological features potentially suggestive of ischemia or infarction. However, MRI neuroimaging studies did not show evidence of any ischemic damage to the right-sided basal ganglia, and was therefore potentially suggestive of antiphospholipid antibodies exerting inflammatory as opposed to thrombotic manifestations^58–60^ (see Discussion Section).

Patient 8 had psoriatic arthritis and was treated with the TNF-inhibitor infliximab, and was diagnosed with ALS after 1 year of treatment. Although presenting with a mixed upper-motor neuron and lower-motor neuron syndrome categorized as ALS, our patient had several clinical, electrodiagnostic, and immunological findings which were highly atypical for ALS. This included acute onset of tetraparesis, clinical and electrodiagnostic evidence of a sensory polyneuropathy, lumbar puncture studies showing an elevated total protein of 117 mg/dl, and demonstration of antibodies against the P/Q-type calcium channel.

## LITERATURE REVIEW

### Purpose of Literature Review

In the presented literature review, we consider whether patients with rheumatic diseases and Parkinsonism have features which are consistent with or discrepant from idiopathic Parkinson's disease. We also consider whether the other movement and neurodegenerative disorders which we defined in our case series were previously described in rheumatic diseases. We additionally highlight features in the literature which afford insight into underlying mechanisms. This approach is highly relevant to similarly understand mechanisms of movement and other neurodegenerative disorders in our patients.

### Search Strategy

To identify such patients, we searched the Medline/PubMed database (National Library of Medicine, Bethesda, MD) using the following Medical Subject Headings (MeSH) search tags: “Movement Disorder,” “Parkinson's Disease,” “Atypical Parkinsonism,” “Parkinsonian,” “Parkinsonian Syndromes,” “Parkinsonism,” “Multiple System Atrophy,” “Multiple System Atrophy-Cerebellar,” “MSA-C,” “Multiple System Atrophy-Parkinsonian,” “MSA-P,” “Olivopontocerebellar Atrophy,” “Shy–Drager Syndrome,” “Striatonigral Degeneration,” “Corticobasal Ganglionic Degeneration,” “Corticobasal Syndrome,” “Cerebellar Degeneration,” “Amyotrophic Lateral Sclerosis,” “ALS,” “Frontotemporal,” “Frontotemporal Dementia,” “Progressive Supranuclear Palsy,” “Ataxia,” “Chorea,” “Dystonia,” “Myoclonus,” “Tics,” “Tremor,” “Sjögren's syndrome,” “Lupus Erythematosus, Systemic,” “Lupus,” “Antiphospholipid syndrome,” “Vasculitis,” “Vasculitis, Central Nervous System,” “Behcet Syndrome,” “Giant Cell Arteritis,” “Takayasu Arteritis,” “Anti-Neutrophil Cytoplasmic Antibody-Associated Vasculitis,” “Wegener's Granulomatosis,” “Granulomatosis with Polyangiitis,” “Microscopic Polyangiitis,” “Scleroderma, Systemic,” “Arthritis, Rheumatoid,” “Arthritis, Psoriatic,” “Ankylosing Spondylitis,” “Spondyloarthropathies,” “Seronegative Spondyloarthropathy,” “TNF-Inhibitor.” We considered patients described in different formats (including case series, case reports, and observational studies), and defined the following inclusion and exclusion criteria:

### Inclusion Criteria

Patients with described clinical features either consistent with Parkinsonian syndromes, movement disorders, and other neurodegenerative syndromes as defined in the Preview Section; *OR* Patients described as having Parkinson's disease, but presenting with a constellation of symptoms, examination findings, and/or neuroimaging findings which ubiquitously cause a wider spectrum of CNS deficits than typically seen in Parkinson's disease. We considered this second criterion as particularly important, given that several of our patients were initially misclassified as having Parkinson's disease (ie, as opposed to Parkinsonian syndromes), and given the likelihood that the rheumatic disease literature may similarly misclassify patients as having Parkinson's disease (ie, instead of Parkinsonian syndromes).

### Exclusion Criteria

Pediatric cases with an age <18 years, given that pediatric patients may have different mechanisms of underlying rheumatic diseases and different mechanisms of movement and other neurodegenerative disorders; studies without sufficient clinical information to characterize the underlying movement or neurodegenerative disorders; studies which were unavailable in the English language; studies which were published before 1980. With regard to this latter criterion, we desired to define rheumatic diseases based on current appreciation about the spectrum of clinical features and associated markers of autoimmunity. In general, criteria before 1980 may not have included antibody markers which are currently included in respective rheumatic diseases such as systemic lupus erythematosus (SLE) (ie, antidouble DNA antibodies and lupus anticoagulant not included before the revised SLE 1982 criteria of Tan et al)^61^; did not necessarily identify and distinguish between rheumatic diseases as distinct entities (ie, primary versus secondary SS), and may not have included clinical features which are now regarded as encompassing the full spectrum of distinct rheumatic diseases.

### Overall Results of Literature Studies

Altogether, we identified 101 potential studies of movement and other neurodegenerative disorders in rheumatic diseases. Of these studies, we excluded 11 studies reporting on pediatric cases^62–72^; 13 reported in non-English languages^73–85^; 6 studies reported before 1980^86–91^; and 4 studies which did not have sufficient clinical information to characterize the underlying movement and neurodegenerative disorders.^92–95^

Therefore, our literature review identified a total of 67 studies describing movement and other neurodegenerative disorders in different rheumatic diseases (Table 2). Clinical features of Parkinsonism and other disorders which were identified in rheumatic disease patients are considered below.

**TABLE 2 (Continued):**
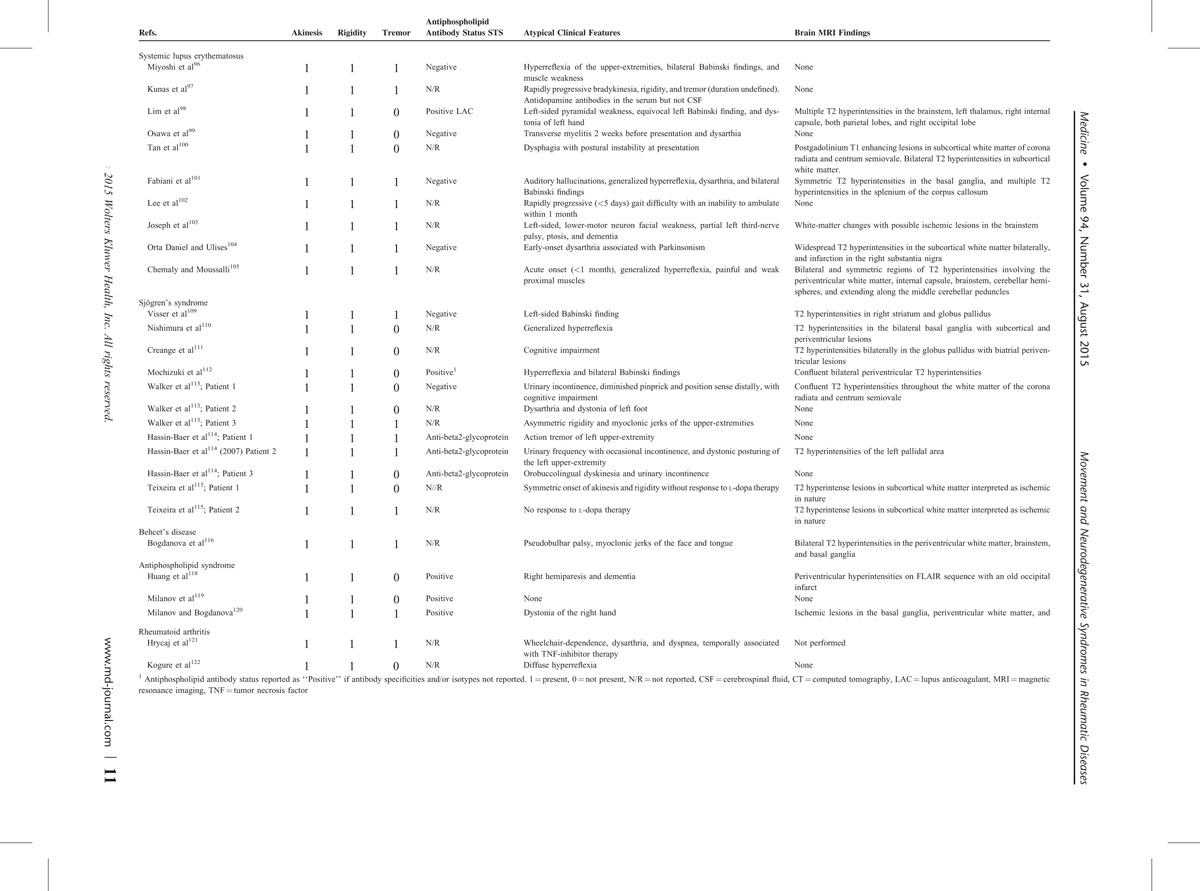
Literature Review of Movement and Other Neurodegenerative Disorders in Rheumatic Diseases

**TABLE 2 (Continued):**
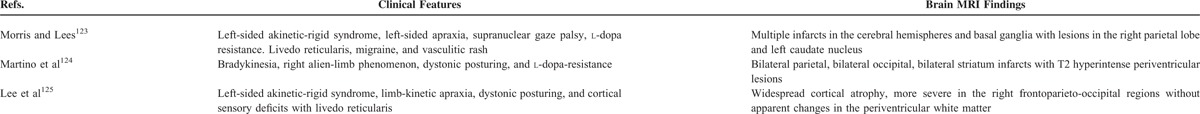
Literature Review of Movement and Other Neurodegenerative Disorders in Rheumatic Diseases

**TABLE 2 (Continued):**
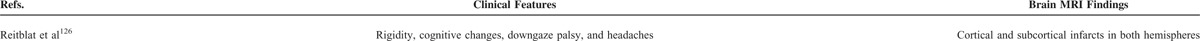
Literature Review of Movement and Other Neurodegenerative Disorders in Rheumatic Diseases

**TABLE 2 (Continued):**
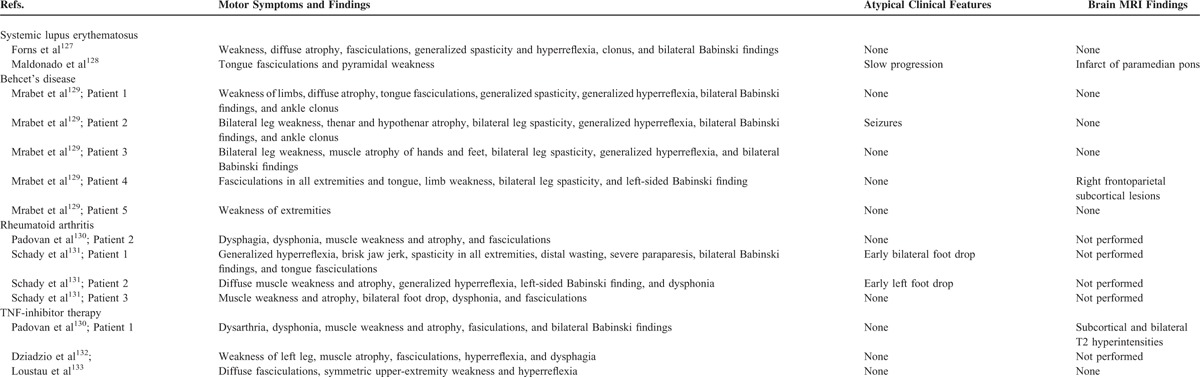
Literature Review of Movement and Other Neurodegenerative Disorders in Rheumatic Diseases

**TABLE 2 (Continued):**
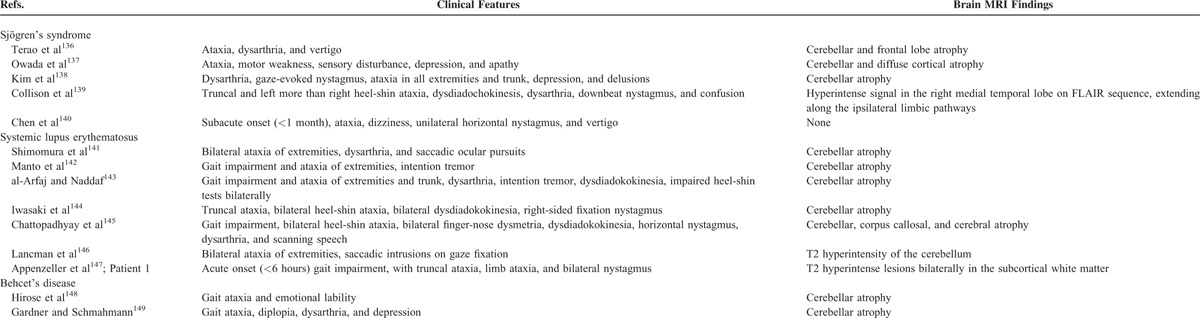
Literature Review of Movement and Other Neurodegenerative Disorders in Rheumatic Diseases

**TABLE 2 (Continued):**
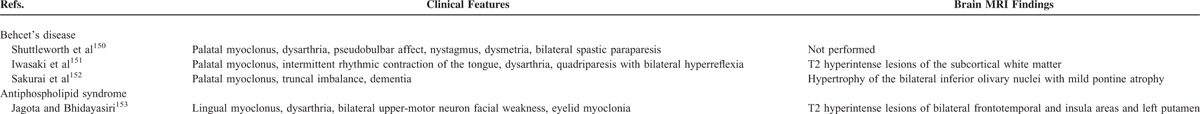
Literature Review of Movement and Other Neurodegenerative Disorders in Rheumatic Diseases

**TABLE 2 (Continued):**
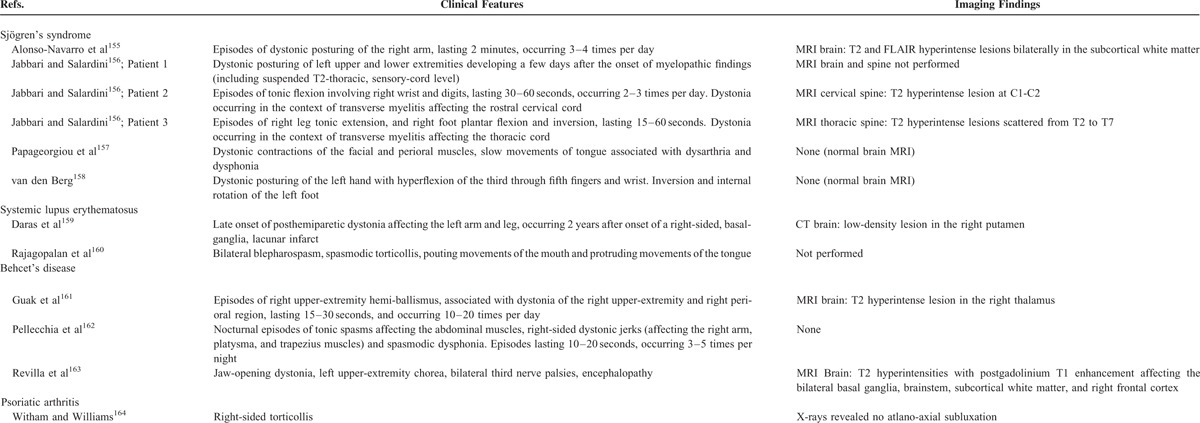
Literature Review of Movement and Other Neurodegenerative Disorders in Rheumatic Diseases

**TABLE 2 (Continued):**
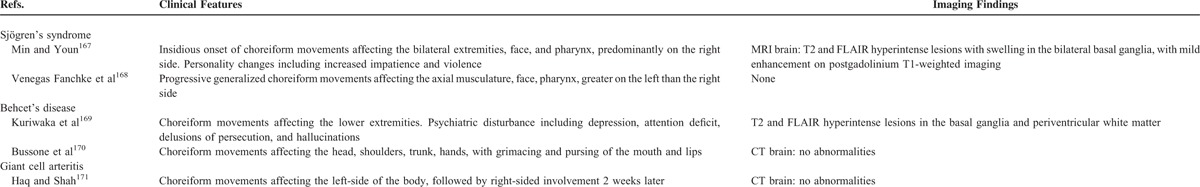
Literature Review of Movement and Other Neurodegenerative Disorders in Rheumatic Diseases

### Parkinsonian Syndromes

#### Systemic Lupus Erythematosus (SLE)

The presentation of Parkinsonian syndromes in rheumatic disease patients is shown in Table 2A. We identified 10 manuscripts describing Parkinsonism in a total of 10 SLE patients.^96–105^ Of these patients, a striking finding was that no patients had features consistent with idiopathic Parkinson's disease. Instead, all patients had clinical and neuroimaging findings demonstrating a more ubiquitous pattern of injury. Clinical features which were inconsistent with idiopathic Parkinson's disease included a rapid onset of symptoms,^97,102,105^ presence of dementia at disease onset,^103^ myalgias and muscle weakness,^96,105^ auditory hallucinations,^101^ and pyramidal/upper-motor neuron findings.^96^

CNS syndromes in SLE may be associated with an underlying vasculopathy or antineuronal antibodies.^106^ Similarly, the described SLE patients with Parkinsonian syndromes also had features suggesting an association with a vasculopathy as well as antineuronal antibodies.

One SLE patient presented with acute onset of Parkinsonism which was suggestive of a stroke-like presentation.^105^ Another patient presented with infarction of the substantia nigra.^104^ This is an example of “vascular” Parkinson's, which can occur when there are strategic lesions causing infarction of the basal ganglia and/or the substantia nigra. Additionally, even when there were no visible ischemic insults on MRI to the basal ganglia or substantia nigra, SLE patients were also reported as having brain MRIs showing changes in anatomic regions targeted in small-vessel ischemic disease. Such radiographic changes included T2 hyperintensities or infarcts affecting the brainstem,^98,103,105^ the cerebellum,^105^ other gray-matter structures,^98^ and the subcortical white-matter.^81,85^ None of the patients with these abnormal brain MRI findings had a clinical presentation consistent with demyelinating disease, and there was only a single patient with transverse myelitis who did not have any abnormalities on brain MRI.^80^

There was a single patient who was reported as having antidopamine antibodies in the serum but not the CSF.^97^ This patient presented with a normal MRI of the brain. Interestingly, in other SLE CNS syndromes which may be associated with antineuronal antibodies (ie, cognitive impairment with anti-NR2 glutamate receptor antibody),^106^ patients may present with normal MRI studies of the brain. Another patient presented with bilateral and symmetric T2 hyperintense lesions affecting the basal ganglia.^101^ It has been hypothesized that the symmetric nature of these basal ganglia lesions reflects injury mediated by antineuronal antibodies.^107^ However, the presence of symmetric T2 hyperintense, basal ganglia lesions can also be associated with vascular injury, as seen in venous infarcts, hypoxic-ischemic injury, and mitochondrial cytopathies.^108^

#### Sjögren's Syndrome

We identified 7 manuscripts describing Parkinsonism in a total of 12 SS patients.^109–115^ Similar to SLE, no patients with SS had a presentation consistent with idiopathic Parkinson's disease. Instead, SS patients presented with a more clinically ubiquitous pattern of findings, including early-onset dystonia/dystonic posturing,^113,114^ early-onset of dysarthria,^113^ and orobuccolingual dyskinesias.^114^

In contrast to SLE, evidence for an underlying vasculopathy was present but less pronounced in SS. Two patients presented with asymmetric lesions affecting the basal ganglia.^109,114^ In the absence of basal ganglia lesions, there were 3 patients who presented with subcortical white-matter changes without clinical evidence of demyelinating disease.^113,115^ However, unlike SLE patients, there were no patients with stroke-like presentations and acute onset of Parkinsonism. Also in contrast to SLE, there were no patients who had lesions in the thalamus, brainstem, or cerebellum.

There were 4 patients who had entirely normal brain MRI studies.^113,114^ Similar to SLE, the presence of normal brain MRIs in patients with clinically diverse patterns of injury may be associated with antineuronal antibodies. There were 2 patients with symmetrical basal ganglia lesions.^110,111^ Although there were 4 patients described as having antiphospholipid antibodies,^112,114^ only a single patient had potential thrombotic disease which affected the left pallidal region.^114^

#### Behcet's Syndrome

Similar to SLE and SS patients, the described Behcet's syndrome patient with Parkinsonism^116^ did not present as having idiopathic Parkinson's disease, and instead had additional features of pseudobulbar palsy with myoclonic jerks of the face and tongue. Although Behcet's syndrome is traditionally considered to cause CNS disease due to demyelinating or ischemic injury,^117^ the described patient did not otherwise present with features of a demyelinating syndrome.

#### Antiphospholipid Syndrome

Of 3 described patients with antiphospholipid syndrome,^118–120^ only a single patient presented with features consistent with “idiopathic” Parkinson's disease.^119^ As expected in antiphospholipid syndrome, 2 patients presented with ischemic changes or infarction on MRI studies.^118,120^ Such ischemic changes were associated with focal findings, including right-sided hemiparesis and right-sided dystonia.

#### Rheumatoid Arthritis

There were 2 patients with rheumatoid arthritis (RA) presenting with Parkinsonism.^121,122^ Similar to SLE, SS, Behcet's syndrome, and antiphospholipid syndrome patients, neither of these patients presented with findings consistent with idiopathic Parkinson's disease. In 1 RA patient, features inconsistent with idiopathic Parkinson's disease included early-onset of upper-motor neuron findings.^103^ Interestingly, the other RA patient developed rapidly progressive Parkinsonism over a 12-month course of TNF-inhibitor therapy (infliximab), culminating with inability to ambulate, severe bulbar dysfunction, and dyspnea.^121^ Given that infliximab otherwise induced remission of her RA, it was suggested that the rapidity of Parkinsonism might be mediated by infliximab.

### Corticobasal Syndrome (CBS) and Progressive Supranuclear Palsy (PSP)

Tables 2B and C show that CBS and PSP were only described in 4 patients with antiphospholipid syndrome,^123–126^ but in no other rheumatic diseases. In 3 patients, MRI neuroimaging studies revealed diffuse thrombotic disease.^104,105,107^ Patients also could have other clinical features seen in antiphospholipid syndrome, including migraines^123^ and a livedo-reticularis rash.^123,125^

### Amyotrophic Lateral Sclerosis (ALS)

Table 2D describes that there were 7 reports of ALS in 14 rheumatic disease patients, including 2 patients with SLE,^127,128^ 5 patients with Behcet's syndrome,^129^ 4 patients with RA who were not on TNF-inhibitor therapy,^130,131^ and 3 patients (1 with ankylosing spondylitis and 2 with RA) with ALS developing in the context of TNF-inhibitor therapy.^130,132,133^ Three patients presented with abnormal MRI findings,^128–130^ but with lesions which were not spatially disseminated, and therefore could not account for a diffuse ALS-plus presentation. Although 2 patients with foot drop were categorized as having atypical clinical findings,^128,129,131^ the initial clinical presentation of ALS is heterogeneous, and can encompass clinical findings such as an early foot drop.^134,135^ Unlike our patient (see case vignette 8), there were otherwise no immunological features which were identified as being incommensurate with idiopathic ALS.

### Ataxia

Ataxia not reflective of a peripheral neuropathy (ie, not presenting as a “sensory” ataxia), and not occurring secondarily to a stroke or seizure, was altogether seen in 14 rheumatic disease patients. As indicated in Table 2E, this included 5 SS patients,^136–140^ 7 SLE patients,^141–147^ and 2 patients with Behcet's syndrome.^148,149^ Similar to our patients, there were 10 patients who had imaging findings of cerebellar degeneration on MRI studies.^136–138,141–145,148,149^ There was a single patient who had normal imaging studies,^140^ and a single patient with T2 hyperintensity affecting the cerebellum.^146^ Interestingly, there was 1 patient who had additional features of a limbic encephalopathy associated with an ataxia.^139^ This patient presented with confusion and MRI showing FLAIR-hyperintense signals which affected the right medial temporal lobe, and also extended into the ipsilateral limbic pathways. In addition, delusions were reported in 1 patient and depression in 3 patients.^137,138,149^

### Myoclonus

Table 2F reveals that myoclonus was altogether detected in 4 patients, including 3 Behcet's syndrome patients,^150–152^ and in 1 patient with antiphospholipid syndrome.^153^ Interestingly, no patients had myoclonus affecting the limb or truncal musculature. Instead, the 3 Behcet's syndrome patients all had palatal myoclonus,^150–152^ and the patient with antiphospholipid syndrome patient had lingual myoclonus.^153^ Palatal myoclonus can reflect brainstem injury.^154^ Two Behcet's syndrome patients presented with clinical findings suggestive of brainstem injury. In these patients, findings of paraparesis or tetraparesis were associated with dysarthria,^150,151^ pseudobulbar affect,^150^ and dysmetria.^150^ One patient had brainstem abnormalities on brain MRI (hypertrophy of the bilateral inferior olivary nuclei with pontine atrophy),^152^ and the other patient only had subcortical white matter disease.^151^ Similarly, the antiphospholipid syndrome patient with lingual myoclonus also had bilateral upper-neuron findings, with more extensive parenchymal disease affecting the frontotemporal and insular regions.^153^

### Dystonia

Table 2G demonstrates that dystonia was collectively described in 12 rheumatic disease patients, including 6 patients with SS,^155–158^ 2 SLE patients,^159,160^ 3 Behcet's syndrome patients,^161–163^ and 1 patient with psoriatic arthritis.^164^ Altogether, the most common pattern was paroxysmal, unilateral limb dystonia seen in 8 patients.^155,156,158,159,161,162^ There were 4 patients who had dystonia affecting the facial or pharyngeal muscles, which could be associated with blepharospasm,^160^ torticollis,^160,164^ tongue dystonia associated with dysarthria,^157^ spasmodic dysphonia,^162^ and jaw-opening dystonia.^163^ Interestingly, limb dystonia developed in 3 SS patients who had clinical features of an inflammatory myelopathy,^156^ with 2 patients having MRIs supportive of transverse myelitis.^156^ In addition, there was 1 SLE patient with imaging evidence of a previous right putaminal infarct who developed a later-onset, hemiparetic dystonia.^159^

### Chorea

As noted in the Introduction Section, the relationship between chorea and SLE and antiphospholipid syndrome has been well established, with reports seen in the literature going back more than 3 decades, and has been the subject of many review articles.^165,166^ Therefore, we focused our literature review on chorea occurring in association with other rheumatic diseases. Table 2H describes that chorea was noted in 5 patients with other rheumatic disorders, including 2 patients with SS,^167,168^ 2 Behcet's syndrome patients,^169,170^ and 1 patient with giant-cell arteritis.^171^ The choreiform movements were diffuse, occurring bilaterally in the extremities in all patients, and also affecting the truncal and facial musculature in 3 patients.^167,168,170^ There were 2 patients who had abnormal MRIs showing bilateral involvement of the basal ganglia.^167,169^ Psychiatric syndromes including impulsivity, delusions, and hallucinations occurred in 2 patients,^167,169^ suggesting a disease process which was more extensive than an extrapyramidal disorder.

### Other Disorders

Of 166 consecutively evaluated patients with giant-cell arteritis who were assessed for neurological findings, there were 6 patients described as having tremor.^172^ Three patients were described as having essential tremor, 1 patient categorized as having a resting tremor, and 2 patients were classified as having a cerebellar tremor. However, specific clinical characteristics of these tremors were not provided. Other reports of tremor in rheumatic disease patients occurred more broadly in the context of definable movement disorders. A 68-year-old female with SLE was described who developed tremor before onset of associated SLE symptoms, and whose tremor was not associated with another movement disorder.^173^

We did not find any manuscripts when the search term “tics” was combined with any of the other search terms used in our literature search.

## SUMMARY OF THE LITERATURE REVIEW

Of the 28 patients who were described as developing Parkinsonism in the context of SLE, SS, antiphospholipid syndrome, Behcet's syndrome, and RA, a particularly notable finding is that only a single patient had features which were consistent with idiopathic Parkinson's disease. Whereas normal MRI studies or symmetric basal ganglia lesions occurring in some SLE patients may suggest the presence of antineuronal antibodies, the larger subset of SLE patients with acute onset of Parkinsonism, vascular Parkinson's, and other radiographic patterns of vascular injury suggest an association with an underlying vasculopathy. Clinical and MRI features of a vasculopathy also occurred in Parkinsonism patients with antiphospholipid syndrome and Behcet's syndrome. Similarly, the described patients with PSP and CBS had clinical and neuroimaging findings which could include diffuse thrombotic disease.

In contrast, a larger subset of SS patients presented with Parkinsonism occurring in the absence of clinical and neuroimaging findings suggestive of a vasculopathy. These findings were consistent with our case series, in which SS patients with Parkinsonism also did not have MRI patterns of an underlying vasculopathy.

The described rheumatic disease patients with ALS predominantly had a typical disease course seen in idiopathic disease, and therefore suggested that ALS was coincidental and not related to the background rheumatic disease. Additionally, clinical findings of cerebellar degeneration which occurred in SS, SLE, and Behcet's patients were uniformly associated with cerebellar degeneration on MRI studies.

Unlike our patients, none of the reported SS patients with any movement or other neurodegenerative syndromes presented with PNS disease associated with DRG neurodegeneration. The propensity of our patients to present with coexisting neurodegenerative syndromes is an additionally unique feature which was not reported in the literature and is further considered below.

## DISCUSSION

We here describe a spectrum of movement and other neurodegenerative syndromes in our rheumatic disease patients. There were several significant findings. First, none of our patients presented with Parkinson's disease, and were instead affected by a variety of significantly less prevalent movement and other neurodegenerative disorders. This finding is consistent with our literature review, which demonstrated that rheumatic disease patients with movement disorders almost never presented with Parkinson's disease. Second, we defined that patients could present with multiple, coexisting movement disorders. Third, to help understand why rheumatic disease patients could present with uncommon and frequently cooccurring movement and neurodegenerative disorders, we have sought to compare potential mechanisms in the literature with our patients. This integration of the literature review with our case series is further discussed below.

The most notable example of how multiple co-occurring syndromes evolved in a single patient was demonstrated in Patient 1. She presented with a sensory neuronopathy, nonlength-dependent, small-fiber neuropathy, cerebellar degeneration, and a progressive nonfluent aphasia with PET findings supportive of FTD (Figure 1A–D). As noted, the probability that a single patient could present with these multiple disorders is on the order of ∼1-per-billion—and therefore suggests that such syndromes may be mechanistically interrelated. Patient 2 is another example of an SS patient with a sensory neuronopathy, non-length-dependent, small-fiber neuropathy, and cerebellar degeneration. Aside from such co-occurring syndromes, other widespread patterns of clinical injury which were seen in our patients included supranuclear gaze palsy, cerebellar ataxia, dystonia, and an alien-limb phenomenon.

Therefore, we reviewed the literature and considered etiopathogenic mechanisms to explain how our patients could present with such widespread clinical deficits. One possibility to explain the widespread pattern of CNS deficits seen in our patients is a commensurately widespread pattern of vasculopathic injury. In our review of the literature, Table 2 indeed illustrates that some patients with SLE, SS, Behcet's, and antiphospholipid syndrome could present with MRI neuroimaging studies suggestive of a vasculopathy. Such associated clinical findings and radiographic patterns suggestive of a vasculopathy included acute onset of symptoms, focal sensorimotor findings suggestive of a stroke, ischemic changes affecting gray-matter structures, as well as subcortical white-matter disease in the absence of demyelinating syndromes.

However, none of our patients presented with MRI neuroimaging studies suggestive of a widespread vasculopathy. Therefore, another potential mechanism to explain the propensity of our patients to present with widespread clinical findings and cooccurring neurodegenerative disorders is the presence of antineuronal antibodies, which may ubiquitously target shared autoantigens dispersed in different PNS and CNS compartments. Careful scrutiny of our patients’ clinical deficits and ancillary studies provides credence to such antineuronal antibody mechanisms.

In this regard, it is of particular interest that Patient 1 and Patient 2, who presented with sensory neuronopathies and cerebellar degeneration, are phenotypically similar to specific paraneoplastic syndromes associated with antineuronal antibodies.^174,175^ In such paraneoplastic disorders, this identical pattern of sensory neuronopathies coupled with cerebellar degeneration is associated with anti-Hu antibodies. Such anti-Hu antibodies target shared neuronal autoantigens which are expressed in both the DRG (ie, associated with a neuronopathy) and the cerebellum. Similarly, sensory neuronopathies and cerebellar degeneration may also occur in association with celiac disease.^37,38^ Our workup did not detect the presence of anti-Hu antibodies. An SS patient with ataxia developing in the context of a limbic encephalopathy was described as having antibodies in the sera which not only immunostained Purkinje cells of the cerebellum, but also neurons in the DRG.^139^ Even though this patient did not have clinical features of a sensory neuronopathy (ie, associated with large-size DRG neurodegeneration), this manuscript further supports our findings suggesting that the co-occurrence of neuronopathies and cerebellar ataxia in a single patient may reflect immune-mediated mechanisms targeting shared autoantigens in the DRG and the cerebellum.^139^

Therefore, the shared phenotype in our patients of sensory neuronopathies coupled with cerebellar degeneration interestingly suggests the presence of novel antibody specificities and shared immune-mediated mechanisms. Therefore, we are now pursuing further studies to evaluate for such novel antibody specificities.

The absence of vascular patterns of damage on our MRI neuroimaging studies also indicates that antibodies associated with our reported movement and neurodegenerative disorders may exert pleiotropic mechanisms. For example, in Patient 7 with UCTD, the presence of a hemi-Parkinsonian syndrome associated with antiphospholipid antibodies was not associated with brain MRI showing any ischemic changes. Interestingly, SS patients have similarly been described who presented with Parkinsonism, anti-beta-2 glycoprotein antibodies, but also without MRI evidence of vascular injury.^114^ In such cases, it has been hypothesized that antiphospholipid antibodies may cross-react with neuronal structures and cause inflammatory as opposed to vasculopathic injury.^58,60^

Patient 3 interestingly illustrates how patients can present with co-occurring disorders which may reflect a continuum of neuropathological changes. In particular, this patient presented with features of CBS (ie, limb apraxia, alien-limb phenomenon) and PSP (ie, supranuclear gaze palsy and early falls within 1 year of symptoms onset). Both of these disorders are associated with abnormal deposition of tau proteins in different parts of the brain, and are both referred to as “tauopathies.”^20,48^ Just as damaged and aggregated proteins may be targeted by autoantibodies, it can be hypothesized that misfolded tau proteins may be a substrate for autoantigens in patients with rheumatic diseases, and may account for overlapping features of CBS and PSP seen in rheumatic diseases.

In addition, just as posttranslational modifications of proteins (including phosphorylation) may be a mechanism leading to autoantibody development, phosphorylated alpha-synuclein (which is deposited as neuronal and glial-cell aggregates in Parkinson's disease and MSA) may represent an analogous type of a neoepitope.^176–178^ Given that our patients could present with PNS disorders before the development of CNS neurodegenerative diseases, skin-biopsies could potentially be used to evaluate whether unmyelinated or autonomic fibers express autoantigens which are shared with the CNS, such as phosphorylated alpha-synuclein.

The presence of antineuronal antibodies may also provide a biomarker that a disorder which is presumptively regarded as noninflammatory may be underscored by immune-mediated mechanisms. Such an example is epitomized in Patient 8, who was diagnosed with the neurodegenerative disorder of ALS in the context of being treated with TNF-inhibitor therapy for psoriatic arthritis. However, this patient had substantive features suggestive of a distinct ALS-plus syndrome which was iatrogenically induced and immune-mediated, including a very rapid progression of tetraparesis (ie, 20 falls developing over a 1-month period), the high total protein on CSF studies in the absence of any structural disease (ie, such as CSF-block due to a disc–osteophyte complex), and with serologies showing antineuronal antibodies against the P/Q-type calcium channel. The latter finding is intriguing given the proclivity of TNF-inhibitors to induce autoantibodies.^179–182^ The spectrum of neurological disorders which may be attributable to TNF-inhibitors is broadening.^179,181^ In other TNF-inhibitor-induced, neurological disorders, there may be improvement, arrest, or further deterioration upon withdrawal of TNF-inhibitors.^179,183^ Our patient demonstrated disease progression despite withdrawal of TNF-inhibitor therapy. We were unable to obtain approval for IVIg or plasmapheresis, and due to functional debility and high-risk of infections (ie, aspiration pneumonia), the patient and her clinicians thought that immunosuppressive therapy carried unacceptable risks.

There have been 3 prior studies of ALS developing in patients treated with TNF-inhibitors.^130,132,133^ Nevertheless, cardinal and unique features in our case—including CSF abnormalities and the presence of antineuronal antibodies—were not described in these 3 other patients. We therefore believe that our patient represents the first case in the literature which provides the most convincing evidence for a TNF-inhibitor-induced ALS-plus syndrome, with several collective features suggestive that the TNF-inhibitors were causative and associated with an immune-mediated syndrome. Additional ALS-plus cases presenting in patients treated with TNF-inhibitor therapy, especially when associated with atypical features, should be reported. In addition, we note that in our study, EMG of the bulbar musculature was not performed. In patients who otherwise present with acute denervation in 3 other body regions (ie, trunk, lower-extremity, and upper-extremity), it may be felt that assessment for bulbar denervation may not provide additional insight. However, we acknowledge that selective sparing of the bulbar musculature in our patient could have constituted an additional “red flag” against the diagnosis of ALS, and provided an additional layer of support for a TNF-induced ALS-plus syndrome. Therefore, we feel that an extensive evaluation for denervation in the bulbar as well as the trunk and limb musculature should be part of the electrodiagnostic evaluation for patients who present with suspected ALS in the context of TNF-inhibitor therapy.

In the differential diagnosis of ALS-plus syndromes, it is important to consider the possibility of an inflammatory process occurring at the level of the nerve roots. Such an inflammatory process can lead to an elevated CSF total protein, and also account for lower-motor neuron findings. On nerve conduction studies, the findings of prolonged F-waves may be an electrophysiological indicator of an inflammatory process affecting the nerve roots, especially in the absence of spinal stenosis, other mechanical causes of a polyradiculopathy, and demyelinating neuropathies.^184^ In this study, F-waves performed in the lower- and upper-extremity were normal. Furthermore, the presence of upper-motor findings would not be expected with an inflammatory process restricted to the nerve roots, and further supports the patient's presentation as being an ALS-plus syndrome. Nevertheless, given the propensity of TNF-inhibitors to induce a wide variety of PNS disorders, assessment for prolonged F-waves should be evaluated in patients presenting with a potential ALS-plus syndrome in the context of TNF-inhibitor therapy.

Altogether, our patients were mainly referred when they were no longer able to independently ambulate, and suffered from infections and other adverse complications stemming from severe deconditioned states (ie, sacral decubitus ulcers). In such scenarios, the risk of immunosuppressive therapy was deemed as being prohibitive. However, several of our patients were initially diagnosed as having Parkinson's disease, but were maintained on l-dopa therapy despite rapid and progressive disease progression. Therefore, earlier recognition of atypical Parkinsonian syndromes in rheumatic disease patients may permit a more opportune and safe therapeutic window to intervene with immunomodulatory therapy.

However, we acknowledge that our study does not directly address whether such immunomodulatory therapy would be beneficial. For example, if immune-mediated injury is only a proximal and initial event which triggers a neurodegenerative disorder, then the enlarging armamentarium of neuroprotective agents and not immunomodulatory therapy would be therapeutically appropriate. In contrast, if degeneration of neuronal structures serves as a substrate for triggering and amplifying neuronal injury (ie, unmasking of novel epitopes), then there may be a preferential niche for immunomodulatory therapy.

Inclusion body myositis (IBM) is a useful model for understanding how a disease may have neurodegenerative as well as immune-mediated mechanisms. In contrast to other inflammatory myopathies (ie, dermatomyositis and polymositis), IBM is associated with earlier onset of distal weakness, increased frequency of bulbar dysfunction, and refractoriness to immunosuppressive therapy.^185^ Recent findings supportive of antigen-driven mechanisms include distinct haplotype associations, intramuscular infiltrates of clonally restricted CD4 and plasma cells,^186^ and the recent finding that ∼1/3 of patients with IBM have antibodies against cytosolic 5′-nucleotidase 1A (cN-1A).^187^

Studies have suggested that SLE and SS patients with IBM may have different profiles compared to IBM patients without rheumatic diseases. Such findings include increased frequency of the HLA-DR3 haplotype in SS^188^ and a higher frequency of SS and rheumatic diseases in IBM compared to the general population.^189^ Interestingly, compared to an ethnically matched control group, IBM patients had a similar frequency of myositis-specific antibodies but a higher frequency of non-organ-specific antibodies as seen in SLE and SS (anti-Ro52 and anti-Ro60 antibodies).^190^ Finally, some but not all studies have reported that the evolution of IBM may differ in SS and SLE patients versus the general population, including increased responsiveness to immunosuppressive therapy.^191,192^

Therefore, such lessons from IBM are analogous and can be extrapolated to our studies, and collectively suggest how movement and neurodegenerative disorders may also be modulated by immune-mediated mechanisms when occurring in patients with rheumatic disease.

We consider it unlikely that the proclivity of our patients to present with disorders other than Parkinson's disease reflects selection bias—especially given that these patients could be referred with an initial diagnosis of Parkinson's disease. This illustrates how Parkinsonian syndromes and the disorders described in this manuscript may be confused with Parkinson's disease. Careful scrutiny for atypical features of Parkinson's disease in rheumatic diseases is therefore especially important.

Given that our study is a case series, larger cohort studies are now warranted to further investigate the spectrum and pathogenesis of movement and other neurodegenerative disorders in rheumatic diseases. Our case series was enriched in patients with SS, and did not include any patients with SLE. However, of the more than 2000 patients with SLE evaluated as part of the Hopkins Lupus Cohort, there are no patients with Parkinsonism or any other movement or neurodegenerative disorders which we reported in our study (Dr. Michelle Petri, personal communication). The current era in which SLE disease activity is aggressively treated in the earliest stages of incipient and evolving disease activity may account for a more restricted spectrum of SLE CNS syndromes.

In summary, we describe rheumatic disease patients with underreported or previously unreported movement and other neurodegenerative syndromes. Our patients had wide-spread patterns of clinical injury more expansive than Parkinson's disease, could present with multiple neurodegenerative disorders, lacked MRI evidence of a vasculopathy, and could share disease phenotypes seen in antibody-associated paraneoplastic syndromes. Further studies are now warranted to further characterize the clinical spectrum and mechanisms of movement and other neurodegenerative disorders in rheumatic diseases.
